# Daily stress and worry are additional triggers of symptom fluctuations in individuals living with Long COVID: results from an intensive longitudinal cohort study

**DOI:** 10.1093/abm/kaaf093

**Published:** 2025-11-12

**Authors:** Daryl B O’Connor, Darren C Greenwood, Maedeh Mansoubi, Nawar D Bakerly, Aishwarya Bhatia, Johnny Collett, Helen E Davies, Joanna Dawes, Brendan C Delaney, Leisle Ezekiel, Phaedra Leveridge, Ghazala Mir, Willie Muehlhausen, Clare Rayner, Janet T Scott, Manoj Sivan, Ian Tucker-Bell, Himanshu Vashisht, Tomás Ward, Darren Winch, Helen Dawes, LOCOMOTION Consortium, Mauricio Barahona, Alexander Casson, Jonathan Clarke, Vasa Curcin, Helen Davies, Carlos Echevarria, Sarah Elkin, Rachael Evans, Zaccheus Falope, Ben Glampson, Trisha Greenhalgh, Stephen Halpin, Mike Horton, Joseph Kwon, Simon de Lusignan, Gayathri Delanerolle, Erik Mayer, Harsha Master, Ruairidh Milne, Jacqui Morris, Amy Parkin, Stavros Petrou, Anton Pick, Nick Preston, Amy Rebane, Emma Tucker, Ana Belen Espinosa Gonzalez, Sareeta Baley, Annette Rolls, Emily Bullock, Megan Ball, Shehnaz Bashir, Joanne Elwin, Denys Prociuk, Iram Qureshi, Samantha Jones

**Affiliations:** School of Psychology, University of Leeds, Leeds, LS2 9JT, United Kingdom; School of Medicine and Leeds Institute for Data Analytics, University of Leeds, Leeds, LS2 9JT, United Kingdom; Medical School, University of Exeter, Exeter, EX1 2HZ, United Kingdom; NIHR Exeter Biomedical Research Center, Medical School, Faculty of Health and Life sciences, University of Exeter, Exeter, EX1 2LU, United Kingdom; Northern Care Alliance, Salford, M6 8HD, United Kingdom; Medical School, University of Exeter, Exeter, EX1 2HZ, United Kingdom; Department of Sport, Health and Social Work, Oxford Brookes University, Oxford, OX3 0BP, United Kingdom; Cardiff and Vale University Health Board, Cardiff, CF14 4TT, United Kingdom; Medical School, University of Exeter, Exeter, EX1 2HZ, United Kingdom; Department of Surgery & Cancer, Imperial College London, London, W12 0NN, United Kingdom; School of Health Sciences, University of Southampton, Southampton, SO17 1BJ, United Kingdom; Medical School, University of Exeter, Exeter, EX1 2HZ, United Kingdom; Leeds Institute for Health Sciences, University of Leeds, Leeds, LS2 9JT, United Kingdom; SAFIRA Clinical Research Limited, Tipperary, E53 VW10, Ireland; Patient Advisory Group (PAG); COVID Recovery Service, NHS Highlands, Raigmore Hospital, Inverness, IV2 3BW, United Kingdom; MRC-University of Glasgow Centre for Virus Research, Glasgow, G61 1QH, United Kingdom; Academic Department of Rehabilitation Medicine, University of Leeds, Leeds, LS2 9JT, United Kingdom; Patient Advisory Group (PAG); SAFIRA Clinical Research Limited, Tipperary, E53 VW10, Ireland; Insight SFI Research Centre for Data Analytics, Dublin City University, Dublin, D04 V1W8, Ireland; Patient Advisory Group (PAG); Medical School, University of Exeter, Exeter, EX1 2HZ, United Kingdom; NIHR Exeter Biomedical Research Center, Medical School, Faculty of Health and Life sciences, University of Exeter, Exeter, EX1 2LU, United Kingdom

**Keywords:** post-COVID condition, stress, physical exertion, mental exertion, symptoms, ecological momentary assessment

## Abstract

**Background:**

Recent research has shown that exertion in physical, cognitive, social, and self-care activities triggers symptom severity in individuals with Long COVID.

**Purpose:**

The current study aimed to investigate whether daily emotional exertions (stress, worry, rumination) were associated with symptom exacerbation, over and above influences of effortful daily activities, in individuals with Long COVID.

**Methods:**

In total, 376 participants were recruited from UK Long COVID clinics and community settings and completed daily assessments of activity and severity of 8 core symptoms every 3 hours for up to 24 days; 155 participants completed daily assessments of stress, worry, and rumination for at least 7 consecutive days.

**Results:**

Days with higher stress scores were associated with increased severity of all symptoms on the same day, after adjusting for activities, demographic and medical factors (*P*-values ≤ .007). Days with higher stress scores also predicted more severe anxiety and depression symptoms 1 day later (*P* < .001) and more severe anxiety (*P* < .001) and dizziness symptoms (*P* = .003) 2 days later. Days with higher worry scores were associated with increased fatigue (*P* < .001), anxiety (*P* < .001), depression (*P* < .001), and cognitive dysfunction (*P* = .002) on the same day, but decreased anxiety (*P* = .003) and depression (*P* = .002) symptoms 1 day later and less severe pain (*P* = .002) symptoms 2 days later. Daily rumination was only associated with 2 symptoms.

**Conclusions:**

Daily stress and worry are distinct factors linked to fluctuations in same-day and next-day Long COVID symptoms, with daily stress showing the strongest association—consistent with patterns of postexertional symptom exacerbation. These findings highlight the importance of considering stress and worry as potential therapeutic targets and integrating their management into self-care programs.

## Introduction

Post-COVID-19 condition, or Long COVID, represents a global health issue. An estimated 10% of individuals who contracted COVID-19 continue to report symptoms lasting beyond 12 weeks.^1-3^ The symptomatology and adverse effects on functioning are significantly debilitating for patients and are similar across high-income and low- to middle-income countries.^4^ Long COVID is not limited to individuals who experienced a severe acute infection or required hospitalization. Symptoms can vary widely but often include fatigue, breathlessness, palpitations, dizziness, pain, cognitive difficulties, anxiety, and depression.^1^^,^^5^ These symptoms may fluctuate throughout the day and from one day to the next within the same individual. A defining feature of Long COVID for many patients is the significant and often unpredictable variation in symptoms over hours and days, as well as the diverse responses to and recovery from potential triggering events.^1^^,^^3^^,^^5-7^

A growing body of work has found that physical exertions are associated with symptom exacerbations.^8^^,^^9^ For example, Burton et al,^9^ using an intensive longitudinal design, found that physical activity was associated with increases in fatigue in individuals with Long COVID. In particular, individuals experiencing a delayed response to activity found it peaked between 1 and 2 days later.^9^ More recently, in a substantially larger study, Greenwood et al^8^ found that exertion in physical, cognitive, social, and self-care activities was associated with increased symptom severity, not only of fatigue and breathlessness shortly afterwards but also a range of symptoms, including dizziness and cognitive dysfunction and that some of the symptom exacerbation was delayed to the next day. These findings highlight the importance of managing all types of effortful activity, not just physical but also cognitive and social activities, and that an individual’s response to that activity may be experienced 1 or 2 days later. However, they also raise the possibility that other factors, such as stress, worry, and rumination, may also trigger symptom exacerbation, in addition to the physical, cognitive and social activities.

Psychological stress arises when an individual perceives a discrepancy, whether real or not, between the demands of a situation and the resources they have to meet these demands.^10^^,^^11^ This subjective evaluation, known as cognitive appraisal, leads to the stress response which includes triggering changes in behavior, affect, and physiological processes. Moreover, psychological stress can affect health outcomes directly, through adversely influencing autonomic and neuroendocrine responses, but also indirectly, through changes in health behaviors.^12^ A related concept which can also influence health is perseverative cognition: the cognitive representation of past stressful events (rumination) or feared future events (worry).^13^ It has been argued that worry, rumination, and related thought processes may influence disease processes by prolonging stress-related physiological activation by amplifying short-term responses, delaying recovery, or reactivating responses after a stressor has been experienced.^13^^,^^14^ More recently, it has been suggested that worry and rumination can also negatively impact on a range of health behaviors that can influence health processes.^15^^,^^16^ Therefore, given that Long COVID is a new condition with an uncertain etiology and prognosis, it is likely that factors such as stress, worry, and rumination about having the condition may play a role in influencing the patient experience and symptom exacerbation.

Living with Long COVID has been shown to be associated with high levels of stress, worry, and uncertainty. Numerous studies have shown that contracting COVID-19 was associated with long-lasting poor mental health^17^ and that levels of COVID-related worry remained high throughout the pandemic.^18^ Moreover, it is well established that psychological stress, worry, and rumination can influence key physiological processes that may affect symptom exacerbation.^12^^,^^14^^,^^19-21^ For example, a recent review has shown that higher levels of rumination are associated with elevations in multiple indicators of inflammation.^20^ Therefore, the aim of the current study was, using data from Greenwood et al,^8^ to investigate the extent to which daily stress, worry, and rumination about COVID-19 were associated with fluctuations in Long COVID symptoms on the same day, 1 and 2 days later, over and above the influences of effortful daily activities. Specifically, we hypothesized that days with greater levels of stress, worry, and rumination would be associated increases in Long COVID symptoms on the same day, 1 and 2 days later (after controlling for the influences of effortful daily activities). Using smartphones to implement intensive longitudinal methods, we examined how various symptoms fluctuate in response to daily assessments of stress, worry, and rumination about COVID. This was conducted over 3 separate 8-day assessment periods, spaced several weeks apart, to embed within analysis a wide range of variation in lived experience and contextual factors in a cohort of individuals living with Long COVID.

## Methods

This intensive longitudinal cohort study is part of the LOCOMOTION research program. LOCOMOTION is a multisite initiative that incorporates technology-assisted monitoring of condition-specific outcome measures. A comprehensive protocol for program of research and this study have been previously published^22^^,^^23^ and is summarized here, emphasizing our primary objective: quantifying the extent to which activities predict subsequent symptoms using ecological momentary assessments (EMAs). This study was co-designed with Long COVID patients to align with their priorities. The LOCOMOTION study included an 8-member patient and public involvement advisory group representing diverse backgrounds. Three members tested study methods, ensuring ease of use, and 2 are co-authors of the work.

Participants aged 18 and older were recruited from 10 Long COVID services within the UK National Health Service as part of the LOCOMOTION Consortium between February 2022 and August 2023. Recruitment was open regardless of hospitalization status or SARS-CoV-2 test results (positive or negative) to allow generalizability to people who were infected before widespread availability of polymerase chain reaction (PCR) or lateral flow tests or presented with milder symptoms from the initial infection. Additionally, a community sample was gathered through general practice networks and social media. Exclusion criteria included an inability to use mobile or wearable technology, language barriers, known pregnancy, or a prior diagnosis of dementia or cognitive impairment.

Upon recruitment, participants provided demographic details, including age, sex, ethnicity, employment status, infection history, and vaccination history. They then completed EMAs at 5 time points throughout the day over 8 consecutive days. This process was repeated for another 8-day period at 6- and 12-week postrecruitment (Figure S1). Participants with fewer than 7 consecutive days of EMA data were excluded. Study data were collected and managed using REDCap electronic data capture tools hosted at the University of Exeter, with EMAs delivered via the AthenaCX platform for mobile phones.

### Ecological momentary assessments

Participants received push notifications on their phones to complete an EMA every 3 hours between 9:00 am and 9:00 pm each day (Figure S1), with responses allowed within a 45-minute window. The EMA was co-designed with Long COVID patients, building on prior research.^9^ Each EMA collected information on the primary activity performed in the past 30 minutes (categorized as physical, cognitive, social, self-care, rest, or sleep), the level of effort required (rated from 0 “no effort” to 10 “most effortful”), the presence of symptoms, and their severity (rated from 0 “no problem” to 10 “severe problem”). The core symptom list was adapted from the COVID-19 Yorkshire Rehabilitation Scale and included fatigue, pain or discomfort, dizziness, palpitations, cognitive dysfunction, anxiety, and depression.^24^ Additionally, each EMA asked participants, “Thinking about the last hour, to what extent have you? 1. Felt stressed, 2. Worried about your illness in the future, and 3. Thought about your illness in the past” (rated on a continuous scale from 0 “Not at all” to 10 “a great deal”). These latter items were based on the UK COVID-19 Mental Health and Well-being Study^18^^,^^25^ which demonstrated good face, current, and predictive validity.

### Statistical analysis

The statistical analysis aimed to estimate potential delayed responses to stress, worry, and rumination experienced by participants the same day, the day before, or 2 days before, recorded using the EMAs. Research questions such as this, generating intensive longitudinal data collected over multiple time points, requires a combination of multilevel modelling (to take account of symptoms within days within participants) and time series analysis (to model the time-lags between exposure triggers and symptom outcomes). A flexible approach to this complex data structure is Markov chain Monte Carlo methods applied within a Bayesian framework. These are similar in concept to random-effects models. Note, missing outcomes (response data) are handled through additional latent variables, which are assumed missing at random with values generated from the posterior predictive distribution, similar to multiple imputation by chained equations. However, the AR1 and AR2 mechanisms (see below) benefitted from consecutive day-level information and any missing time-level EMA data were removed by collapsing over the day, so there were no missing response data.

The epidemiological exposures were feeling stressed, worried about their illness in the future, and thoughts about their illness in the past (rumination). These exposures were used to predict the severity of 8 self-reported symptoms which were modelled as joint multivariate outcomes. Both exposures and outcomes were recorded using the EMAs and scored 0-10. Scores were aggregated over the day for ease of computation and to facilitate estimation of delayed responses to triggers over the following days. The model took account of the multilevel structure within joint multivariate symptoms within days nested within participants. Correlations between symptom severity scores on consecutive days were incorporated using autoregressive time-series of order 1 (AR1), and modelling stress, worry, and rumination on preceding days used autoregressive time-series of order 2 (AR2) to allow for any delayed responses.

Models adjusted for age (years), sex (female, male), minority ethnicity (yes, no), employment status (full-time, part-time, other), location (clinic or community setting), preexisting autoimmune condition (yes, no), preexisting mental health condition (yes, no), whether initially asymptomatic with COVID-19 infection (yes, no), whether the participant was hospitalized (yes, no), admitted to intensive care unit (yes, no), dominant variant at infection (original, alpha, delta, omicron), whether completed 2 vaccinations before initial infection (yes, no), duration of Long COVID at the start of the study, and mean efforts in physical, cognitive, social, and self-care activities scored 0-10 over the previous days (AR2). All continuous covariates were grand mean-centered to reduce collinearity and improve interpretation of coefficients.

Subgroup analyses were conducted to investigate whether associations between stress, worry, and rumination and symptom severities were modified by gender (male, female), and separately by having a preexisting mental health condition (yes or no). Effect modification was formally modelled by including the appropriate interaction terms in the model and joint testing of both the linear and nonlinear components of the restricted cubic splines.

All models were carried out in JAGS 4.3.0, managed by the runjags package in R (version 4.3.1), using the High Performance Computing facilities at the University of Leeds.

### Sample size

This intensive longitudinal cohort study was originally aimed to recruit enough participants to analyze approximately 300 participants, providing 80% power to detect a 20% improvement in fatigue over 12 weeks in 1 of 3 equally sized groups of participants relative to another. However, the current analysis required complete data over consecutive days, with subsequent exclusions resulting in fewer participants available for analysis. Resulting credible intervals allow estimation of adjusted mean symptom severity scores to within less than ±1 point on the 0-10 scale for all symptoms, and in most cases with substantially greater precision, demonstrating adequate sample size for estimating all associations of interest.

## Results

### Recruitment

Out of 514 participants who were approached (351 from clinics, 163 community), 420 (82%) consented to participate in the study (301 from clinics, 119 community). A total of 376 (73%) provided symptom data (273 from clinics, 103 community), and 155 (41%) of these completed questionnaires on stress, worry, and rumination for at least 7 consecutive days (105 from clinics, 50 community). Of the 155 included, 90 provided responses over 7 days, 52 over 14 days, and 13 over 21 days, generating 1631 days of EMA data. Mean (SD) age of participants was 48 (12) years, 114 (74%) were female, with a median (interquartile range) duration of Long COVID of 15 months (9-24) at the time of recruitment (Table 1). Only 26 (17%) reported a preexisting mental health condition prior to their COVID-19 infection, and just 66 (43%) were in full-time employment at the time of recruitment (Table 1). Intraclass correlations (within-participants, between-days) were moderately high (Table S1).

**Table 1.** Characteristics of participants from Long COVID clinic and community samples.

	Long COVID clinic patients	Community-based	Total
	(*n* = 105)	(*n* = 50)	(*n* = 155)
**Mean age (years)(SD)**	47 (12)	50 (10)	48 (12)
**Female gender (%)**	76 (72%)	38 (76)	114 (74)
**Ethnicity (%)**			
** White**	93 (89)	46 (92)	139 (90)
** Black**	0 (0)	1 (2)	1 (1)
** Asian**	4 (4)	0 (0)	4 (3)
** Mixed/other**	8 (8)	3 (6)	11 (7)
**Employment status (%)**			
** Full-time**	50 (48)	16 (32)	66 (43)
** Part-time**	21 (20)	8 (16)	29 (19)
** Self-employed**	9 (9)	1 (2)	10 (6)
** Not in paid employment**	25 (24)	25 (50)	50 (32)
** Not recorded**	0 (0)	0 (0)	0 (0)
**Preexisting comorbidities (%)**			
** Allergies or autoimmune conditions**	15 (14)	12 (24)	27 (17)
** Other respiratory conditions**	0 (0)	0 (0)	0 (0)
** Other inflammatory conditions**	2 (2)	0 (0)	2 (1)
** Hypertension**	2 (2)	1 (2)	3 (2)
** Hypotension**	0 (0)	0 (0)	0 (0)
** Other heart conditions**	3 (3)	3 (6)	6 (4)
** Type 2 diabetes mellitus**	1 (1)	0 (0)	1 (1)
** Mental health condition**	21 (20)	5 (10)	26 (17)
**Completed vaccinations before initial infection (%)**	66 (63)	24 (48)	90 (58)
**Dominant variant at time of infection (%)**			
** Original**	32 (30)	22 (44)	54 (35)
** Alpha**	4 (4)	2 (4)	6 (4)
** Delta**	23 (22)	7 (14)	30 (19)
** Omicron**	46 (44)	19 (38)	65 (42)
**Asymptomatic with initial infection (%)**	5 (5)	0 (0)	5 (3)
**Admitted to hospital with initial infection (%)**	8 (8)	2 (4)	10 (6)
**Admitted to intensive care with initial infection (%)**	2 (2)	0 (0)	2 (1)
**Median duration of Long COVID (IQR) (months)**	12 (8-18)	19 (13-35)	15 (9-24)
**Clinic location (%)**			
** Birmingham**	5 (5)	-	-
** Cardiff**	19 (18)	-	-
** Hertfordshire**	14 (13)	-	-
** NHS Highland**	2 (2)	-	-
** Imperial College London**	4 (4)	-	-
** Leeds**	5 (5)	-	-
** Leicester**	4 (4)	-	-
** Newcastle**	5 (5)	-	-
** Oxford**	33 (31)	-	-
** Salford**	14 (13)	-	-

### Descriptive statistics

Mean observed symptom severity scores (ranging from 0 to 10) are presented in Table 2. Levels of daily stress and worry regarding one’s illness were higher than daily thoughts about the illness in the past, averaged over the days. Mean scores for symptoms indicated that participants reported the highest severity for fatigue, followed by pain or discomfort. Men reported higher average symptom scores than women across most domains, including stress, worry, rumination, anxiety, and depression.

**Table 2.** Mean observed symptom severity scores (0-10), by participant characteristics, with 95% confidence intervals.

	Stress	Worry	Rumination	Breathlessness	Fatigue	Pain/discomfort	Dizziness	Palpitations	Anxiety	Depression	Cognitive dysfunction
**All participants**	2.1 (1.8-2.4)	2.0 (1.7-2.3)	1.3 (1.0-1.6)	0.9 (0.7-1.1)	5.4 (5.1-5.8)	2.7 (2.3-3.1)	0.8 (0.6-1.1)	0.4 (0.3-0.5)	1.6 (1.3-1.9)	1.1 (0.8-1.4)	3.0 (2.6-3.4)
**Age (years)**											
** <40**	2.2 (1.6-2.9)	2.0 (1.3-2.7)	1.4 (0.6-2.1)	1.1 (0.6-1.6)	5.6 (5.1-6.2)	2.3 (1.6-3.0)	1.1 (0.5-1.8)	0.4 (0.2-0.6)	1.7 (0.9-2.4)	1.3 (0.7-1.9)	2.9 (2.1-3.8)
** 40-49**	1.9 (1.4-2.3)	1.4 (1.0-1.9)	0.8 (0.5-1.1)	0.9 (0.5-1.3)	5.3 (4.6-6.0)	3.2 (2.4-4.1)	0.7 (0.4-1.1)	0.4 (0.2-0.7)	1.3 (0.8-1.9)	0.7 (0.3-1.1)	3.2 (2.5-4.0)
** 50-59**	2.3 (1.8-2.8)	2.3 (1.7-2.8)	1.7 (1.2-2.2)	0.8 (0.5-1.1)	5.3 (4.7-5.9)	2.8 (2.2-3.5)	0.7 (0.4-1.0)	0.3 (0.1-0.5)	1.7 (1.2-2.3)	1.5 (0.9-2.1)	2.9 (2.2-3.6)
** 60+**	1.7 (1.0-2.3)	2.3 (1.4-3.1)	1.2 (0.5-1.9)	1.0 (0.4-1.5)	5.8 (4.9-6.7)	1.8 (1.1-2.6)	1.1 (0.4-1.7)	0.4 (0.1-0.6)	1.5 (0.8-2.2)	0.6 (0.0-1.3)	2.7 (1.7-3.7)
**Gender:**											
** Female**	1.9 (1.6-2.2)	1.7 (1.3-2.0)	1.1 (0.8-1.4)	0.7 (0.5-0.8)	5.5 (5.1-5.9)	3.0 (2.5-3.4)	0.7 (0.5-1.0)	0.3 (0.2-0.5)	1.4 (1.0-1.7)	0.9 (0.6-1.2)	2.8 (2.3-3.2)
** Male**	2.6 (2.0-3.2)	3.0 (2.2-3.7)	2.0 (1.3-2.6)	1.5 (1.0-2.1)	5.2 (4.5-5.9)	2.0 (1.3-2.8)	1.2 (0.7-1.7)	0.5 (0.2-0.7)	2.1 (1.4-2.7)	1.9 (1.1-2.6)	3.5 (2.7-4.3)
**Employment status:**											
** Full-time**	1.7 (1.3-2.1)	1.7 (1.3-2.2)	1.2 (0.8-1.6)	0.8 (0.5-1.0)	4.9 (4.3-5.5)	2.3 (1.7-2.9)	0.6 (0.3-0.8)	0.2 (0.1-0.3)	1.3 (0.9-1.7)	0.8 (0.4-1.2)	2.4 (1.8-2.9)
** Part-time**	2.7 (2.0-3.3)	2.2 (1.5-2.9)	1.6 (0.9-2.2)	1.0 (0.6-1.4)	5.6 (5.0-6.2)	2.9 (2.1-3.8)	0.8 (0.4-1.3)	0.4 (0.2-0.6)	1.8 (1.1-2.5)	1.3 (0.7-1.9)	3.3 (2.6-4.0)
** Other**	2.2 (1.7-2.7)	2.2 (1.6-2.8)	1.2 (0.8-1.7)	1.0 (0.6-1.5)	6.0 (5.5-6.5)	3.1 (2.4-3.7)	1.2 (0.8-1.7)	0.6 (0.3-0.9)	1.7 (1.1-2.3)	1.4 (0.8-2.0)	3.5 (2.7-4.3)
**Source of participants:**											
** Clinics**	2.2 (1.8-2.6)	2.1 (1.7-2.5)	1.3 (0.9-1.6)	0.8 (0.6-1.1)	5.3 (4.9-5.7)	2.7 (2.2-3.2)	0.8 (0.5-1.0)	0.2 (0.1-0.4)	1.8 (1.4-2.2)	1.2 (0.8-1.6)	2.8 (2.4-3.3)
** Community**	1.9 (1.4-2.4)	1.8 (1.3-2.4)	1.4 (0.9-1.8)	1.0 (0.6-1.4)	5.7 (5.1-6.3)	2.8 (2.1-3.4)	1.0 (0.6-1.4)	0.6 (0.4-0.9)	1.2 (0.7-1.6)	1.0 (0.5-1.5)	3.2 (2.5-4.0)

### Effects of daily stress, worry, and rumination on symptom fluctuations

**Figure 1.**
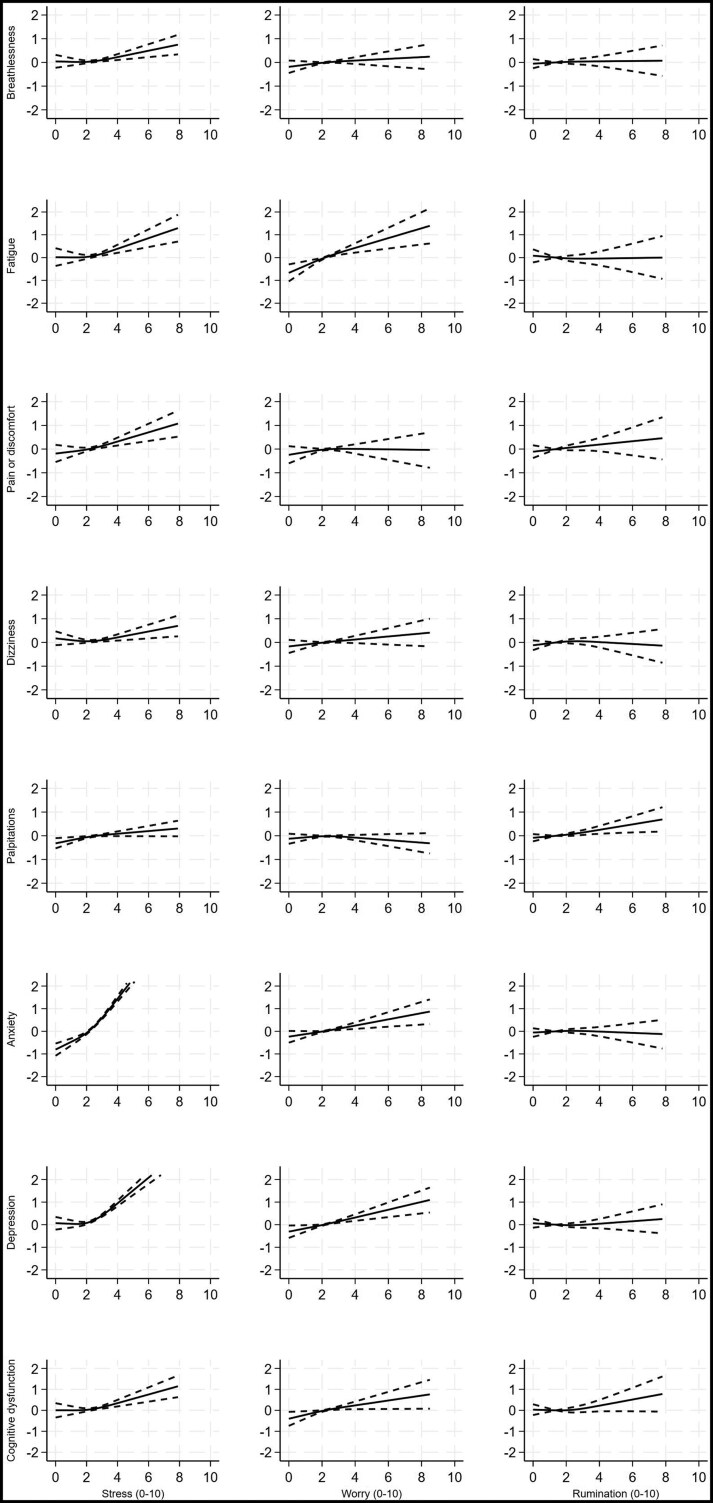
Change in mean symptom severity scores associated with stress (column 1), worry (column 2), and rumination (column 3), on the same day, with 95% credible intervals. Symptoms severity is truncated at the 99th centile for the purposes of presentation. Models adjusted for age, sex, ethnicity, employment status, setting, preexisting conditions, severity of infection, hospitalization, intensive care unit admission, COVID-19 variant, vaccination status, duration of Long COVID, and efforts in physical, cognitive, social and self-care activities (see Table 3 for significance levels).

**Figure 2.**
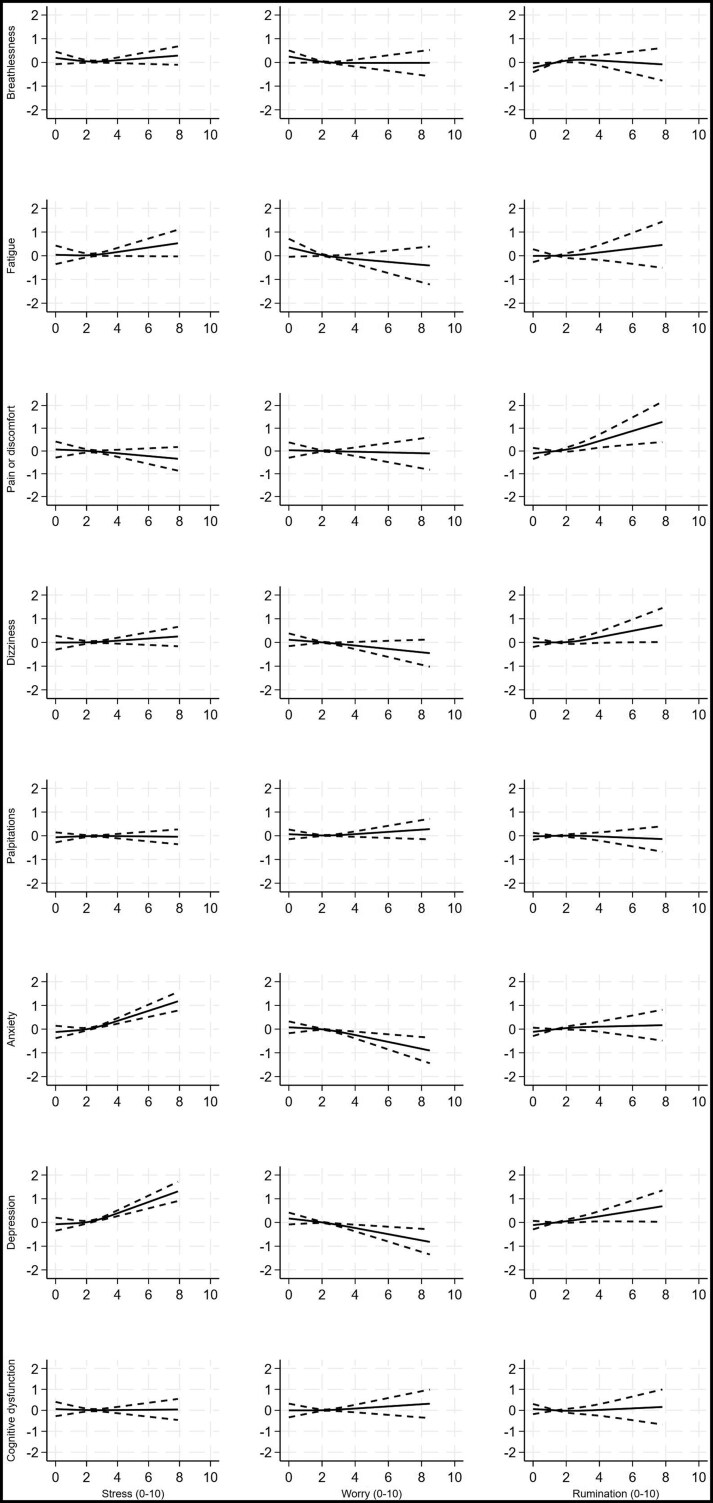
Change in mean symptom severity scores associated with stress (column 1), worry (column 2), and rumination (column 3), 1 day earlier, with 95% credible intervals. Symptoms severity is truncated at the 99th centile for the purposes of presentation. Models adjusted for age, sex, ethnicity, employment status, setting, preexisting conditions, severity of infection, hospitalization, intensive care unit admission, COVID-19 variant, vaccination status, duration of Long COVID, and efforts in physical, cognitive, social and self-care activities (see Table 3 for significance levels).

**Figure 3.**
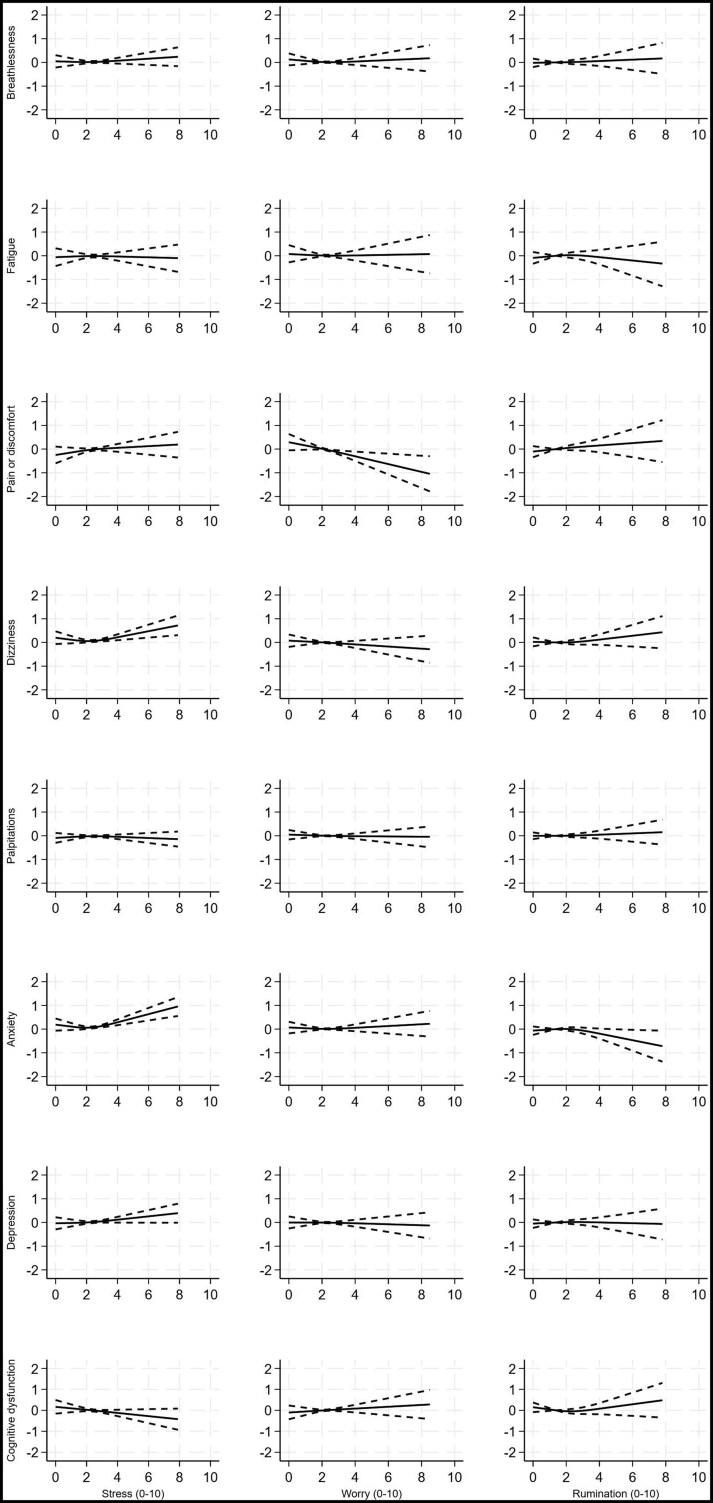
Change in mean symptom severity scores associated with stress (column 1), worry (column 2), and rumination (column 3), 2 days earlier, with 95% credible intervals. Symptoms severity is truncated at the 99th centile for the purposes of presentation. Models adjusted for age, sex, ethnicity, employment status, setting, preexisting conditions, severity of infection, hospitalization, intensive care unit admission, COVID-19 variant, vaccination status, duration of Long COVID, and efforts in physical, cognitive, social and self-care activities (see Table 3 for significance levels).

Models showed that days with higher stress scores were significantly associated with increased severity of *all* symptoms on the same day, after adjusting for effortful activities, demographic and medical factors (all *P*-values ≤ .007; Figure 1, Table 3). The strongest associations were with anxiety (high stress of 8/10 associated with anxiety 4.9 points higher the same day than when experiencing mean stress levels [95% CI, 4.5-5.4; *P* < .001]), and depression (3.2 points higher [95% CI, 2.8-3.6; *P* < .001]) (Table S2). Days with higher stress scores also predicted more severe anxiety (1.2 points higher; 95% CI, 0.8-1.6; *P* < .001) and depression symptoms 1 day later (1.2 points higher; 95% CI, 0.8-1.6; *P* < .001). Participants also reported more severe anxiety (1.0 points higher; 0.6-1.4; *P* < .001) and dizziness symptoms (0.7 points higher; 0.3-1.2; *P* = .003) 2 days later (Figures 2 and 3, Tables S3 and S4).

**Table 3.** Significance levels (*P*-values) for the contribution of each psychological measure (daily stress, worry, and rumination) in the model for each symptom/outcome.^a^

Psychological measure		Same day	1-day time-lag	2-day time-lag
**Daily stress**	Breathlessness	**.001**	.195	.498
	Fatigue	**<.001**	.175	.920
	Pain	**<.001**	.341	.199
	Dizziness	**.007**	.461	**.003**
	Palpitations	**<.001**	.825	.556
	Anxiety	**<.001**	**<.001**	**<.001**
	Depression	**<.001**	**<.001**	.114
	Cognitive dysfunction	**<.001**	.944	.083
**Daily worry**	Breathlessness	.189	.162	.557
	Fatigue	**<.001**	.076	.917
	Pain	.415	.929	**.002**
	Dizziness	.118	.171	.473
	Palpitations	.236	.407	.846
	Anxiety	**<.001**	**.003**	.655
	Depression	**<.001**	**.002**	.902
	Cognitive dysfunction	**.002**	.649	.524
**Daily rumination**	Breathlessness	.839	.073	.861
	Fatigue	.841	.657	.631
	Pain	.384	**.012**	.498
	Dizziness	.508	.136	.448
	Palpitations	**.013**	.836	.852
	Anxiety	.803	.389	.083
	Depression	.625	.057	.867
	Cognitive dysfunction	.189	.830	.261

a Models adjusted for age, sex, ethnicity, employment status, setting, preexisting conditions, severity of infection, hospitalization, intensive care unit admission, COVID-19 variant, vaccination status, duration of Long COVID, and efforts in physical, cognitive, social and self-care activities. Note p-values in bold are statistically significant.

Days with higher worry scores were associated with increased severity of fatigue. Worry of 8/10 was associated with 1.3 points higher fatigue on the same day compared to with mean worry scores (95% CI, 0.6-2.0; *P* < .001), 0.8 points higher anxiety (0.3-1.3; *P* < .001), 1.0 points higher depression (0.5-1.5; *P* < .001), and 0.7 points higher cognitive dysfunction (0.1-1.3; *P* = .002). However, participants also reported 0.8 points less anxiety (−1.3 to −0.3; *P* = .003) and depression (−1.2 to −0.3; *P* = .002) symptoms 1 day later and 1.0 less severe pain (−1.6 to −0.3; *P* = .002) symptoms 2 days later. There was only evidence that daily rumination was associated with 2 symptoms across all the analyses.

### Subgroup analyses

When the models were re-run to investigate whether associations between stress, worry, and rumination and symptom severities were modified by gender (male, female), and separately by having a preexisting mental health condition (yes or no). The results showed that, with a small number of exceptions, the conclusions were broadly consistent across all subgroups (Tables S5 and S6).

## Discussion

This intensive longitudinal study investigated the extent to which daily stress, worry, and rumination were associated with fluctuations in Long COVID symptoms on the same day, 1 and 2 days later, over and above the influences of effortful daily activities and demographic and medical factors. Using smartphones to implement intensive longitudinal methods, our study showed, for the first time, that emotional exertions—daily stress and worry about one’s illness—were unique factors associated with increased severity of same-day Long COVID symptoms. We discovered that feeling stressed was the strongest additional predictor of changes in Long COVID symptoms, with exacerbation in all 8 symptoms on the same day. Increases in stress and worry were associated with changes in multiple symptom domains including breathlessness, dizziness, depression, and cognitive dysfunction. We also found that higher levels of daily stress predicted greater symptom severity for anxiety and depression the following day, along with increased dizziness and anxiety symptoms 2 days later. In contrast, higher levels of worry were associated with reduced symptom severity for anxiety and depression 1 day later, and lower pain severity 2 days later. Finally, subgroup analyses showed that these results were substantively the same in males and females as well as among individuals living with and without a preexisting mental health condition.

The current findings add to an earlier study by Greenwood et al,^8^ which utilized data from the same cohort, revealing that all forms of exertion, whether cognitive, social, self-care, or physical activities, contributed to symptom exacerbation. Here, we identify stress and worry about one’s illness as further triggers or emotional exertions that lead to changes in symptom severity. A recent qualitative investigation into the triggers and symptoms of Long COVID also recognized mental factors, including stress and worry, as significant triggers of symptom exacerbation,^26^ with one patient (59 years old) stating, “If I’m worried about the Long COVID or worried about anything else, that definitely, I can absolutely say that makes it worse.”^26^ These findings are consistent with the growing body of work that has shown that a large range of different types of exertions can lead to postexertional malaise or postexertional symptom exacerbation. A recent systematic review revealed that the prevalence of postexertional malaise in community-dwelling adults living with Long COVID was 25% and that there is an urgent need for more inclusive and rigorous research.^27^

Stress and worry are important variables that psychologically “tax” individuals and can get “under the skin” of individuals producing cumulative chronic stress burden in the longer term.^12^^,^^28^ Moreover, as outlined earlier, stress and worry have been shown to trigger activation of key physiological processes (eg, inflammation, hypothalamic-pituitary adrenal axis functioning) as well as maladaptive behavioral pathways (eg, poor sleep quality, diet, substance use) that can influence symptom exacerbation.^12^^,^^14-16^ For example, it has been suggested that sensitivity of immune cells to glucocorticoids and catecholamines may be the missing link in elucidating how stress may lead to chronic fatigue.^29^ However, the precise pathways linking stress and worry about one’s illness to changes in symptom severity in Long COVID patients remain unknown.

Our finding that worrying about one’s illness predicted decreased anxiety, depression, and pain symptom severity on following days was an unexpected finding. Nevertheless, it suggests that daily stress and worry (about one’s illness) may operate differently over time. Daily stress consistently has a negative impact on symptoms on the same day and on subsequent days. This is in line with recent theorizing that has suggested that the experience of multiple daily stressors can lead to “pileup” on the same day and subsequent days that can impact psychological, affective, physiological, and/or behavioral outcomes.^30^ However, in contrast, there is evidence that engaging in higher levels of worry yielded beneficial effects on the following days. A likely explanation for this is that worrying about one’s illness may trigger engagement in protective coping behaviors and changes in emotional regulation that lead to symptom reduction. For example, worrying about one’s illness may prompt individuals to rest and to disengage in activities that lead to symptom exacerbation. Moreover, this is consistent with a body of work that draws a distinction between excessive general worry and disease-specific worry.^31-33^ An early study by McCaul et al^33^ found that women who had greater worry about breast cancer, even those with the highest levels of worry, were significantly more likely to have performed a breast self-examination, had a mammography screening, and had a clinical breast examination. Furthermore, the idea that higher levels of disease-specific worry are associated with an increase in health protective action is confirmed in a number of reviews and meta-analyses.^31^^,^^32^ Consequently, viewed within the lens of this body of work, our findings relating to the beneficial effects of disease-specific worry on symptom exacerbation are not that surprising, although, it would be important to replicate these findings in a future study.

It was notable that daily rumination about one’s illness had a limited impact on same day and following day symptoms. This was surprising as rumination, as well as worry, has been reliably found to influence somatic symptoms and health-related physiological processes.^12^^,^^14^^,^^21^ One possible explanation for this finding is that disease-specific worries loomed larger in individual’s minds than disease-specific ruminative thoughts due to the uncertainty and unknowns surrounding Long COVID as a new condition. More generally, during the COVID-19 pandemic, COVID-related worries were shown to be much more frequent compared to COVID-related negative repetitive thoughts triggered by past COVID-related events (ie, rumination).^18^ COVID-related worries were also found to be associated with poorer mental health, particularly in individuals living with a mental health condition, whereas COVID-related rumination had less of an impact on mental health measures such as anxiety and depression.^18^ Therefore, although the precise explanation is unclear, the limited effects of rumination compared to worry on symptom fluctuations in individuals living with Long COVID are likely, in part at least, to reflect the uncertain nature of the condition.

Although research into Long COVID is expanding, its precise pathophysiological mechanisms remain unclear and vary widely.^34^^,^^35^ Current theories propose a multifactorial basis involving immune system dysregulation, lingering viral presence, endothelial damage, autonomic nervous system and interoceptive imbalances, mitochondrial dysfunction, and atypical inflammatory responses.^6^^,^^7^^,^^34^^,^^35^ Emerging data suggest that Long COVID is not a single condition, but rather an umbrella term that includes several subtypes, with symptoms clustering into distinct patterns.^35^ Some patients experience postviral fatigue resembling myalgic encephalomyelitis/chronic fatigue syndrome, while others continue to suffer from respiratory or cardiovascular problems. This complexity highlights the importance of personalized treatment plans over a universal therapeutic approach.^22^^,^^36^ A significant obstacle in managing Long COVID is the absence of standardized treatment guidelines. Due to the wide range of symptoms and underlying causes, a multidisciplinary strategy is often advocated—incorporating rehabilitation, lifestyle changes, psychological support, and regular symptom tracking.^5^ Therefore, within this context, our study’s findings suggest that stress and worry related to one’s illness should be included as important additional therapeutic targets. Incorporating stress management into self-care programs may enhance outcomes for individuals living with Long COVID. McCarrick et al^37^ have identified a broad variety of interventions that can reliably reduce worry such as action planning and cognitive behavioral therapy-based approaches. It is likely that acceptance and commitment-based approaches will also yield benefit for stress and worry.^38^ However, the current findings suggest that interventions aimed an intervening at the daily level may be a fruitful way forward. Just-in-time stress management interventions delivered using mobile health approaches have been shown to be effective.^39-41^ For example, Smyth and Heron^39^ found that participants who received random reminders to use stress management skills reported stressful events less frequently, lower stress severity, less negative affect, and exhibited lower levels of the stress hormone cortisol. Therefore, future research ought to also explore the effectiveness of different just-in-time stress management interventions within the context of developing individualized self-care programs to help reduce symptom exacerbation in individuals living with Long COVID.

We recognize that there are some limitations with the current study. First, the main analysis is based on 155 of the 376 participants who provided symptom data. This was because we only included participants who had completed at least 7 consecutive days of daily stress, worry, and rumination measures. This ensured that we had a sufficient number of days to model fluctuations in daily stress, worry, and rumination and to test delayed effects of the predictor variables on subsequent consecutive days. Second, we also acknowledge that we elected to not correct for multiple comparisons. The primary reasons for this decision were because: (1) this is one of the first intensive longitudinal, EMA studies in this patient group and therefore, we wanted the study to be hypothesis-generating with a focus on identifying possible signals; (2) within this context, applying strict multiple-comparison corrections are usually overly conservative and potentially obscure meaningful patterns between the daily triggers and the symptoms, and (3) we were keen to present the unadjusted *P*-values to ensure full transparency. Third, we had low minority ethnic representation, reflecting low representation across UK Long COVID clinics generally. Nevertheless, we did adjust for ethnicity in our analyses, and preliminary analyses found no evidence of differences in symptom reporting or stress, worry, or rumination between White and minority ethnic groups. An adequately powered sample is needed, however, to confirm this finding.^42^ Fourth, participants from Long COVID clinics may not reflect all individuals with Long COVID, and the community-based participants may be more self-selected and not fully representative. Despite these differences, findings were generally consistent across both groups.

The current study also has a number of notable strengths. This study represents one of the most detailed investigations of fluctuations in symptom severity in individuals living with Long COVID while also tracking changes in activity levels, stress, worry, and rumination. It utilized an intensive longitudinal design and included a large sample of participants from Long COVID clinics and community settings over an extended study period ensuring the capture of their lived experience. The EMA methods allowed the recording of predictors, symptoms, and other outcomes close in time to when they occurred, and before some outcomes over the following days are known, thereby improving accuracy and precision and reducing the potential for recall bias. The analyses also controlled for a large range of medical and demographic factors, including severity of the COVID-19 infection, as well as mean efforts on physical, cognitive, social, and self-care activities over the previous days, thereby ruling out key confounding variables.

In conclusion, this study used smartphone-based methods to examine how daily stress, worry, and rumination relate to Long COVID symptom severity. It found that stress and worry about one’s illness—especially stress—were strong unique predictors of increased symptom severity on the same and next day, across various symptoms like breathlessness, dizziness, depression, and cognitive dysfunction. These effects were consistent across gender and mental health history. Integrating stress management into self-care program could improve outcomes for people with Long COVID.

## Supplementary Material

kaaf093_Supplementary_Data

## Transparency statements

The study and analysis plan were preregistered at the ISRCTN registry registered prior to beginning data collection (https://www.isrctn.com/ISRCTN15022307). Requests for deidentified participant data may be submitted to the authors. Applications, including a full research proposal and confirmation of appropriate ethical approval, will be evaluated for compatibility with general objectives, ethical approvals, existing informed consent received, and for potential overlap with ongoing work. Analytic codes used to conduct the analyses presented in this study are not available in a public archive. They may be available by emailing D.C.G. Materials used to conduct the study are not publicly available as they were delivered using an app. However, the items used are clearly described in the article.
