# Supplementary material for: Daily stress and worry are additional triggers of symptom fluctuations in individuals living with Long COVID: results from an intensive longitudinal cohort study
Source: Ann Behav Med. 2025 Nov 12;59(1):kaaf093. doi: 10.1093/abm/kaaf093 (PMC12613253; doi:10.1093/abm/kaaf093)
Supplement: kaaf093_Supplementary_Data [file kaaf093_supplementary_data.zip › Supplemental material ACCEPTED.docx]

**Supplemental material**

| List of consortium members | 2 |
| --- | --- |
| Supplemental Figure 1. Schedule of ecological momentary assessments recording stress, worry and rumination alongside symptom severities throughout the study | 3 |
| Supplemental Table 1. Intraclass correlation coefficients for symptom severity scores (within-participants, between-days) | 4 |
| Supplemental Table 2. Mean change in mean symptom severity scores associated with stress (0-10), by length of time-lag, with 95% credible intervals | 5 |
| Supplemental Table 3. Mean change in mean symptom severity scores associated with worry (0-10), by length of time-lag, with 95% credible intervals | 6 |
| Supplemental Table 4. Mean change in mean symptom severity scores associated with rumination (0-10), by length of time-lag, with 95% credible intervals | 7 |
| Supplemental Table 5. Significance levels (p-values) for tests of interaction terms between psychological measures (stress, worry and rumination) and gender | 8 |
| Supplemental Table 6. Significance levels (p-values) for tests of interaction terms between psychological measures (stress, worry and rumination) and a pre-existing mental health condition | 9 |
|  |  |

**List of consortium members**

Nawar Bakerly, Kumaran Balasundaram, Megan Ball, Mauricio Barahona, Alexander Casson, Jonathan Clarke, Karen Cook, Rowena Cooper, Vasa Curcin, Julie Darbyshire, Helen E Davies, Helen Dawes, Simon de Lusignan, Brendan Delaney, Carlos Echevarria, Sarah Elkin, Ana Belen Espinosa Gonzalez, Rachael Evans, Sophie Evans, Zacchaeus Falope, Ben Glampson, Madeline Goodwin, Trish Greenhalgh, Darren C Greenwood, Stephen Halpin, Juliet Harris, Will Hinton, Mike Horton, Samantha Jones, Joseph Kwon, Cassie Lee, Ashliegh Lovett, Mae Mansoubi, Victoria Masey, Harsha Master, Erik Mayer, Bernardo Meza-Torres, Ruairidh Milne, Ghazala Mir, Jacqui Morris, Adam Mosley, Jordan Mullard, Daryl O'Connor, Rory O'Connor, Thomas Osborne, Amy Parkin, Stavros Petrou, Anton Pick, Denys Prociuk, Clare Rayner, Amy Rebane, Natalie Rogers, Janet T Scott, Manoj Sivan, Adam B Smith, Nikki Smith, Emma Tucker, Ian Tucker-Bell, Paul Williams, Darren Winch, Conor Wood.

**Supplemental Figure 1. Schedule of ecological momentary assessments recording stress, worry and rumination alongside symptom severities throughout the study.** Measures are averaged over times within each day.

**
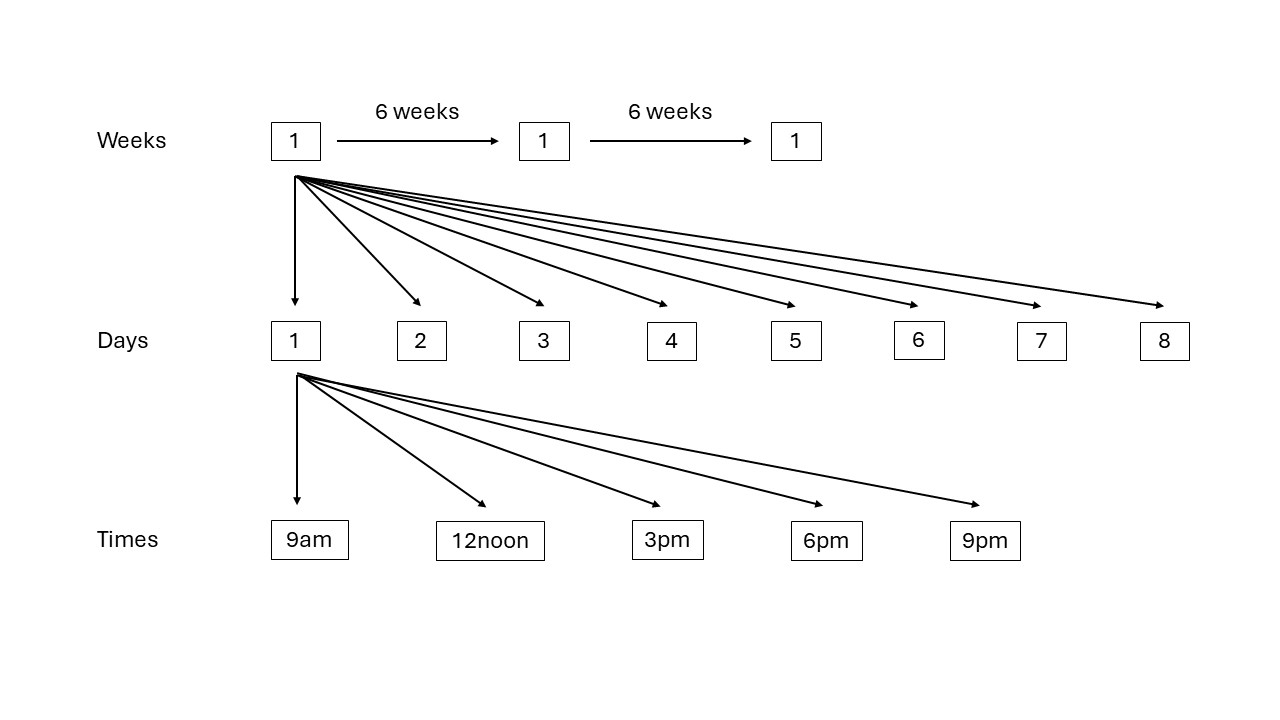
**

**Supplemental Table 1.** Intraclass correlation coefficients for symptom severity scores (within-participants, between-days)

| Symptom severity  (scored 0-10) |  | Intraclass correlation (95% CI) |
| --- | --- | --- |
|  |  |  |
| breathlessness |  | 0.39 (0.31, 0.47) |
| fatigue |  | 0.57 (0.50, 0.64) |
| pain |  | 0.59 (0.52, 0.67) |
| dizziness |  | 0.36 (0.27, 0.47) |
| palpitations |  | 0.28 (0.20, 0.37) |
| anxiety |  | 0.50 (0.42, 0.59) |
| depression |  | 0.52 (0.43, 0.61) |
| cognitive dysfunction |  | 0.66 (0.59, 0.72) |
|  |  |  |
|  |  |  |

**Supplemental Table 2.** Mean change in mean symptom severity scores associated with stress (0-10), by length of time-lag, with 95% credible intervals

| Stress (0-10) | Breathlessness | Fatigue | Pain / discomfort | Dizziness | Palpitations | Anxiety | Depression | Cognitive dysfunction |
| --- | --- | --- | --- | --- | --- | --- | --- | --- |
|  |  |  |  |  |  |  |  |  |
| *Same day* |  |  |  |  |  |  |  |  |
| 0 | 0.0 (-0.2, 0.3) | 0.0 (-0.4, 0.4) | -0.2 (-0.5, 0.2) | 0.2 (-0.1, 0.5) | -0.3 (-0.5, -0.1) | -0.8 (-1.1, -0.5) | 0.1 (-0.2, 0.3) | 0.0 (-0.3, 0.3) |
| 1 | 0.0 (-0.1, 0.2) | 0.0 (-0.2, 0.2) | -0.1 (-0.3, 0.1) | 0.1 (-0.1, 0.3) | -0.2 (-0.3, -0.1) | -0.5 (-0.6, -0.3) | 0.0 (-0.1, 0.2) | 0.0 (-0.2, 0.2) |
| 2.4 (reference) | - | - | - | - | - | - | - | - |
| 4 | 0.2 (0.1, 0.4) | 0.4 (0.2, 0.6) | 0.3 (0.2, 0.5) | 0.2 (0.1, 0.3) | 0.1 (-0.0, 0.2) | 1.4 (1.3, 1.6) | 0.9 (0.8, 1.1) | 0.3 (0.2, 0.5) |
| 8 | 0.8 (0.3, 1.2) | 1.3 (0.7, 1.9) | 1.1 (0.5, 1.7) | 0.7 (0.3, 1.2) | 0.3 (-0.0, 0.6) | 4.9 (4.5, 5.4) | 3.2 (2.8, 3.6) | 1.2 (0.6, 1.7) |
|  |  |  |  |  |  |  |  |  |
| *1 day before* |  |  |  |  |  |  |  |  |
| 0 | 0.2 (-0.1, 0.5) | 0.0 (-0.4, 0.4) | 0.1 (-0.3, 0.4) | -0.0 (-0.3, 0.3) | -0.1 (-0.3, 0.1) | -0.1 (-0.4, 0.1) | -0.1 (-0.3, 0.2) | 0.1 (-0.3, 0.4) |
| 1 | 0.1 (-0.0, 0.3) | 0.0 (-0.2, 0.2) | 0.0 (-0.2, 0.2) | -0.0 (-0.2, 0.2) | -0.0 (-0.2, 0.1) | -0.1 (-0.2, 0.1) | -0.0 (-0.2, 0.1) | 0.0 (-0.2, 0.2) |
| 2.4 (reference) | - | - | - | - | - | - | - | - |
| 4 | 0.1 (-0.0, 0.2) | 0.2 (-0.0, 0.3) | -0.1 (-0.3, 0.1) | 0.1 (-0.0, 0.2) | -0.0 (-0.1, 0.1) | 0.3 (0.2, 0.5) | 0.4 (0.3, 0.5) | 0.0 (-0.1, 0.2) |
| 8 | 0.3 (-0.1, 0.7) | 0.5 (-0.0, 1.1) | -0.3 (-0.9, 0.2) | 0.3 (-0.2, 0.7) | -0.0 (-0.4, 0.3) | 1.2 (0.8, 1.6) | 1.3 (0.9, 1.7) | 0.0 (-0.5, 0.6) |
|  |  |  |  |  |  |  |  |  |
| *2 days before* |  |  |  |  |  |  |  |  |
| 0 | 0.0 (-0.2, 0.3) | -0.1 (-0.4, 0.3) | -0.2 (-0.6, 0.1) | 0.2 (-0.1, 0.5) | -0.1 (-0.3, 0.1) | 0.2 (-0.1, 0.4) | -0.0 (-0.3, 0.2) | 0.2 (-0.2, 0.5) |
| 1 | 0.0 (-0.1, 0.2) | -0.0 (-0.3, 0.2) | -0.1 (-0.3, 0.1) | 0.1 (-0.0, 0.3) | -0.1 (-0.2, 0.1) | 0.1 (-0.0, 0.3) | -0.0 (-0.2, 0.1) | 0.1 (-0.1, 0.3) |
| 2.4 (reference) | - | - | - | - | - | - | - | - |
| 4 | 0.1 (-0.0, 0.2) | -0.0 (-0.2, 0.1) | 0.1 (-0.1, 0.2) | 0.2 (0.1, 0.3) | -0.0 (-0.1, 0.1) | 0.3 (0.2, 0.4) | 0.1 (-0.0, 0.2) | -0.1 (-0.3, 0.0) |
| 8 | 0.2 (-0.2, 0.6) | -0.1 (-0.7, 0.5) | 0.2 (-0.4, 0.7) | 0.7 (0.3, 1.2) | -0.1 (-0.5, 0.2) | 1.0 (0.6, 1.4) | 0.4 (-0.0, 0.8) | -0.4 (-0.9, 0.1) |
|  |  |  |  |  |  |  |  |  |

*Note*: Models adjusted for age, sex, ethnicity, employment status, setting, pre-existing conditions, severity of infection, hospitalisation, intensive care unit admission, COVID-19 variant, vaccination status, duration of Long COVID, and efforts in physical, cognitive, social and self-care activities

**Supplemental Table 3.** Mean change in mean symptom severity scores associated with worry (0-10), by length of time-lag, with 95% credible intervals

| Worry (0-10) | Breathlessness | Fatigue | Pain / discomfort | Dizziness | Palpitations | Anxiety | Depression | Cognitive dysfunction |
| --- | --- | --- | --- | --- | --- | --- | --- | --- |
|  |  |  |  |  |  |  |  |  |
| *Same day* |  |  |  |  |  |  |  |  |
| 0 | -0.2 (-0.4, 0.1) | -0.7 (-1.0, -0.3) | -0.2 (-0.6, 0.1) | -0.2 (-0.4, 0.1) | -0.1 (-0.3, 0.1) | -0.2 (-0.5, 0.0) | -0.3 (-0.6, -0.0) | -0.4 (-0.7, -0.1) |
| 1 | -0.1 (-0.2, 0.0) | -0.4 (-0.6, -0.2) | -0.1 (-0.3, 0.1) | -0.1 (-0.2, 0.1) | -0.1 (-0.2, 0.0) | -0.1 (-0.3, 0.0) | -0.2 (-0.3, -0.0) | -0.2 (-0.4, -0.0) |
| 2.1 (reference) | - | - | - | - | - | - | - | - |
| 4 | 0.1 (-0.1, 0.2) | 0.4 (0.2, 0.6) | 0.0 (-0.2, 0.2) | 0.1 (-0.0, 0.3) | -0.1 (-0.2, 0.0) | 0.3 (0.1, 0.4) | 0.3 (0.2, 0.5) | 0.2 (0.1, 0.4) |
| 8 | 0.2 (-0.3, 0.7) | 1.3 (0.6, 2.0) | -0.0 (-0.7, 0.6) | 0.4 (-0.2, 0.9) | -0.3 (-0.7, 0.1) | 0.8 (0.3, 1.3) | 1.0 (0.5, 1.5) | 0.7 (0.1, 1.3) |
|  |  |  |  |  |  |  |  |  |
| *1 day before* |  |  |  |  |  |  |  |  |
| 0 | 0.2 (-0.0, 0.5) | 0.4 (-0.0, 0.7) | 0.0 (-0.3, 0.4) | 0.1 (-0.2, 0.4) | 0.1 (-0.1, 0.3) | 0.1 (-0.2, 0.3) | 0.2 (-0.1, 0.4) | -0.0 (-0.3, 0.3) |
| 1 | 0.1 (-0.0, 0.3) | 0.2 (-0.0, 0.4) | 0.0 (-0.2, 0.2) | 0.1 (-0.1, 0.2) | 0.0 (-0.1, 0.1) | 0.0 (-0.1, 0.2) | 0.1 (-0.0, 0.2) | -0.0 (-0.2, 0.2) |
| 2.1 (reference) | - | - | - | - | - | - | - | - |
| 4 | -0.0 (-0.2, 0.1) | -0.1 (-0.3, 0.1) | -0.0 (-0.2, 0.2) | -0.1 (-0.3, 0.0) | 0.1 (-0.0, 0.2) | -0.2 (-0.4, -0.1) | -0.2 (-0.4, -0.1) | 0.1 (-0.1, 0.3) |
| 8 | -0.0 (-0.5, 0.5) | -0.4 (-1.1, 0.4) | -0.1 (-0.8, 0.6) | -0.4 (-0.9, 0.1) | 0.3 (-0.1, 0.7) | -0.8 (-1.3, -0.3) | -0.8 (-1.2, -0.3) | 0.3 (-0.3, 0.9) |
|  |  |  |  |  |  |  |  |  |
| *2 days before* |  |  |  |  |  |  |  |  |
| 0 | 0.1 (-0.1, 0.4) | 0.1 (-0.3, 0.4) | 0.3 (-0.1, 0.6) | 0.1 (-0.2, 0.3) | 0.1 (-0.2, 0.2) | 0.1 (-0.2, 0.3) | -0.0 (-0.3, 0.3) | -0.1 (-0.4, 0.2) |
| 1 | 0.1 (-0.1, 0.2) | 0.0 (-0.1, 0.2) | 0.2 (-0.0, 0.3) | 0.0 (-0.1, 0.2) | 0.0 (-0.1, 0.1) | 0.0 (-0.1, 0.2) | -0.0 (-0.1, 0.1) | -0.1 (-0.2, 0.1) |
| 2.1 (reference) | - | - | - | - | - | - | - | - |
| 4 | 0.0 (-0.1, 0.2) | 0.0 (-0.2, 0.2) | -0.3 (-0.5, -0.1) | -0.1 (-0.2, 0.1) | -0.0 (-0.1, 0.1) | 0.1 (-0.1, 0.2) | -0.0 (-0.2, 0.1) | 0.1 (-0.1, 0.3) |
| 8 | 0.2 (-0.3, 0.7) | 0.1 (-0.7, 0.8) | -1.0 (-1.6, -0.3) | -0.3 (-0.8, 0.3) | -0.0 (-0.4, 0.4) | 0.2 (-0.3, 0.7) | -0.1 (-0.6, 0.4) | 0.3 (-0.4, 0.9) |
|  |  |  |  |  |  |  |  |  |

*Note*: Models adjusted for age, sex, ethnicity, employment status, setting, pre-existing conditions, severity of infection, hospitalisation, intensive care unit admission, COVID-19 variant, vaccination status, duration of Long COVID, and efforts in physical, cognitive, social and self-care activities

**Supplemental Table 4.** Mean change in mean symptom severity scores associated with rumination (0-10), by length of time-lag, with 95% credible intervals

| Rumination (0-10) | Breathlessness | Fatigue | Pain / discomfort | Dizziness | Palpitations | Anxiety | Depression | Cognitive dysfunction |
| --- | --- | --- | --- | --- | --- | --- | --- | --- |
|  |  |  |  |  |  |  |  |  |
| *Same day* |  |  |  |  |  |  |  |  |
| 0 | -0.1 (-0.2, 0.1) | 0.1 (-0.2, 0.4) | -0.1 (-0.4, 0.2) | -0.1 (-0.3, 0.1) | -0.1 (-0.2, 0.1) | -0.1 (-0.2, 0.1) | 0.1 (-0.1, 0.3) | 0.0 (-0.2, 0.3) |
| 1.4 (reference) | - | - | - | - | - | - | - | - |
| 2 | 0.0 (-0.0, 0.1) | -0.0 (-0.1, 0.1) | 0.0 (-0.0, 0.1) | 0.0 (-0.0, 0.1) | 0.0 (-0.0, 0.1) | 0.0 (-0.0, 0.1) | -0.0 (-0.1, 0.1) | 0.0 (-0.1, 0.1) |
| 4 | 0.0 (-0.2, 0.3) | -0.0 (-0.3, 0.3) | 0.2 (-0.1, 0.5) | 0.0 (-0.2, 0.2) | 0.3 (0.1, 0.4) | -0.0 (-0.2, 0.2) | 0.0 (-0.2, 0.3) | 0.2 (-0.1, 0.5) |
| 8 | 0.1 (-0.6, 0.7) | 0.0 (-1.0, 1.0) | 0.5 (-0.5, 1.4) | -0.1 (-0.9, 0.6) | 0.7 (0.2, 1.2) | -0.1 (-0.8, 0.5) | 0.3 (-0.4, 0.9) | 0.8 (-0.1, 1.7) |
|  |  |  |  |  |  |  |  |  |
| *1 day before* |  |  |  |  |  |  |  |  |
| 0 | -0.2 (-0.4, -0.0) | -0.0 (-0.3, 0.3) | -0.1 (-0.4, 0.1) | 0.0 (-0.2, 0.2) | -0.0 (-0.2, 0.1) | -0.1 (-0.3, 0.1) | -0.1 (-0.3, 0.1) | 0.1 (-0.2, 0.3) |
| 1.4 (reference) | - | - | - | - | - | - | - | - |
| 2 | 0.1 (0.0, 0.1) | 0.0 (-0.1, 0.1) | 0.1 (-0.0, 0.1) | 0.0 (-0.1, 0.1) | 0.0 (-0.0, 0.1) | 0.0 (-0.0, 0.1) | 0.1 (-0.0, 0.1) | -0.0 (-0.1, 0.1) |
| 4 | 0.1 (-0.1, 0.3) | 0.1 (-0.2, 0.5) | 0.4 (0.1, 0.7) | 0.2 (-0.0, 0.5) | -0.0 (-0.2, 0.1) | 0.1 (-0.1, 0.3) | 0.3 (0.0, 0.5) | 0.0 (-0.3, 0.3) |
| 8 | -0.1 (-0.8, 0.6) | 0.5 (-0.5, 1.5) | 1.3 (0.4, 2.2) | 0.8 (0.0, 1.5) | -0.1 (-0.7, 0.4) | 0.2 (-0.5, 0.8) | 0.7 (0.0, 1.4) | 0.2 (-0.7, 1.0) |
|  |  |  |  |  |  |  |  |  |
| *2 days before* |  |  |  |  |  |  |  |  |
| 0 | -0.0 (-0.2, 0.2) | -0.1 (-0.3, 0.2) | -0.1 (-0.3, 0.1) | 0.0 (-0.2, 0.2) | -0.0 (-0.1, 0.1) | -0.1 (-0.2, 0.1) | -0.0 (-0.2, 0.1) | 0.1 (-0.1, 0.4) |
| 1.4 (reference) | - | - | - | - | - | - | - | - |
| 2 | 0.0 (-0.1, 0.1) | 0.0 (-0.1, 0.1) | 0.0 (-0.0, 0.1) | 0.0 (-0.1, 0.1) | 0.0 (-0.0, 0.1) | 0.0 (-0.1, 0.1) | 0.0 (-0.0, 0.1) | -0.0 (-0.1, 0.0) |
| 4 | 0.1 (-0.2, 0.3) | -0.1 (-0.4, 0.2) | 0.2 (-0.1, 0.4) | 0.1 (-0.1, 0.3) | 0.0 (-0.1, 0.2) | -0.2 (-0.4, 0.0) | 0.0 (-0.2, 0.2) | 0.1 (-0.2, 0.3) |
| 8 | 0.2 (-0.5, 0.9) | -0.3 (-1.3, 0.6) | 0.4 (-0.6, 1.3) | 0.4 (-0.3, 1.2) | 0.2 (-0.4, 0.7) | -0.7 (-1.4, -0.1) | -0.1 (-0.7, 0.6) | 0.5 (-0.4, 1.4) |
|  |  |  |  |  |  |  |  |  |

*Note*: Models adjusted for age, sex, ethnicity, employment status, setting, pre-existing conditions, severity of infection, hospitalisation, intensive care unit admission, COVID-19 variant, vaccination status, duration of Long COVID, and efforts in physical, cognitive, social and self-care activities

**Supplementary Table 5.** Significance levels (p-values) for tests of interaction terms between psychological measures (stress, worry and rumination) and gender

| Psychological measure for which interaction with gender is included | Symptom severity |  | Same  day |  | 1-day time-lag |  |  | 2-day time-lag |  |
| --- | --- | --- | --- | --- | --- | --- | --- | --- | --- |
|  |  |  |  |  |  |  |  |  |  |
| Stress | breathlessness |  | p=0.165 |  | p=0.950 |  |  | p=0.493 |  |
|  | fatigue |  | p=0.699 |  | p=0.478 |  |  | p=0.090 |  |
|  | pain |  | p=0.761 |  | p=0.099 |  |  | p=0.326 |  |
|  | dizziness |  | p=0.566 |  | p=0.568 |  |  | p=0.310 |  |
|  | palpitations |  | p=0.174 |  | p=0.314 |  |  | p=0.287 |  |
|  | anxiety |  | p=0.623 |  | p=0.940 |  |  | p=0.697 |  |
|  | depression |  | p=0.991 |  | p=0.597 |  |  | p=0.077 |  |
|  | cognitive dysfunction |  | p=0.922 |  | p=0.983 |  |  | p=0.009 |  |
|  |  |  |  |  |  |  |  |  |  |
|  |  |  |  |  |  |  |  |  |  |
| Worry | breathlessness |  | p=0.034 |  | p=0.169 |  |  | p=0.869 |  |
|  | fatigue |  | p=0.611 |  | p=0.541 |  |  | p=0.322 |  |
|  | pain |  | p=0.627 |  | p=0.923 |  |  | p=0.030 |  |
|  | dizziness |  | p=0.053 |  | p=0.671 |  |  | p=0.028 |  |
|  | palpitations |  | p=0.875 |  | p=0.565 |  |  | p=1.000 |  |
|  | anxiety |  | p=0.413 |  | p=0.288 |  |  | p=0.458 |  |
|  | depression |  | p=0.831 |  | p=0.313 |  |  | **p=0.001** |  |
|  | cognitive dysfunction |  | p=0.342 |  | p=0.174 |  |  | p=0.424 |  |
|  |  |  |  |  |  |  |  |  |  |
|  |  |  |  |  |  |  |  |  |  |
| Rumination | breathlessness |  | p=0.941 |  | p=0.146 |  |  | p=0.376 |  |
|  | fatigue |  | p=0.711 |  | p=0.389 |  |  | p=0.160 |  |
|  | pain |  | p=0.735 |  | p=0.046 |  |  | p=0.932 |  |
|  | dizziness |  | **p=0.007** |  | p=0.273 |  |  | p=0.786 |  |
|  | palpitations |  | **p=0.001** |  | p=0.026 |  |  | p=1.000 |  |
|  | anxiety |  | p=0.470 |  | p=0.120 |  |  | p=0.310 |  |
|  | depression |  | p=0.367 |  | p=0.904 |  |  | **p=0.004** |  |
|  | cognitive dysfunction |  | p=0.926 |  | p=0.648 |  |  | p=0.197 |  |
|  |  |  |  |  |  |  |  |  |  |

*Note*: Models adjusted for age, sex, ethnicity, employment status, setting, pre-existing conditions, severity of infection, hospitalisation, intensive care unit admission, COVID-19 variant, vaccination status, duration of Long COVID, and efforts in physical, cognitive, social and self-care activities

**Supplementary Table 6.** Significance levels (p-values) for tests of interaction terms between psychological measures (stress, worry and rumination) and a pre-existing mental health condition

| Psychological measure for which interaction with pre-existing mental health condition is included | Symptom severity |  | Same  day |  | 1-day time-lag |  |  | 2-day time-lag |  |
| --- | --- | --- | --- | --- | --- | --- | --- | --- | --- |
|  |  |  |  |  |  |  |  |  |  |
| Stress | breathlessness |  | p=0.169 |  | p=0.955 |  |  | p=0.560 |  |
|  | fatigue |  | p=0.667 |  | p=0.445 |  |  | p=0.102 |  |
|  | pain |  | p=0.803 |  | p=0.094 |  |  | p=0.266 |  |
|  | dizziness |  | p=0.609 |  | p=0.621 |  |  | p=0.344 |  |
|  | palpitations |  | p=0.165 |  | p=0.294 |  |  | p=0.262 |  |
|  | anxiety |  | p=0.590 |  | p=0.921 |  |  | p=0.699 |  |
|  | depression |  | p=0.983 |  | p=0.539 |  |  | p=0.102 |  |
|  | cognitive dysfunction |  | p=0.932 |  | p=0.998 |  |  | p=0.011 |  |
|  |  |  |  |  |  |  |  |  |  |
|  |  |  |  |  |  |  |  |  |  |
| Worry | breathlessness |  | p=0.043 |  | p=0.133 |  |  | p=0.865 |  |
|  | fatigue |  | p=0.552 |  | p=0.512 |  |  | p=0.238 |  |
|  | pain |  | p=0.583 |  | p=0.924 |  |  | p=0.028 |  |
|  | dizziness |  | p=0.067 |  | p=0.703 |  |  | p=0.023 |  |
|  | palpitations |  | p=0.869 |  | p=0.530 |  |  | p=0.996 |  |
|  | anxiety |  | p=0.420 |  | p=0.266 |  |  | p=0.528 |  |
|  | depression |  | p=0.821 |  | p=0.268 |  |  | **p=0.001** |  |
|  | cognitive dysfunction |  | p=0.383 |  | p=0.181 |  |  | p=0.386 |  |
|  |  |  |  |  |  |  |  |  |  |
|  |  |  |  |  |  |  |  |  |  |
| Rumination | breathlessness |  | p=0.942 |  | p=0.164 |  |  | p=0.407 |  |
|  | fatigue |  | p=0.720 |  | p=0.346 |  |  | p=0.167 |  |
|  | pain |  | p=0.829 |  | p=0.049 |  |  | p=0.946 |  |
|  | dizziness |  | **p=0.007** |  | p=0.320 |  |  | p=0.793 |  |
|  | palpitations |  | **p=0.001** |  | p=0.026 |  |  | p=0.996 |  |
|  | anxiety |  | p=0.431 |  | p=0.126 |  |  | p=0.324 |  |
|  | depression |  | p=0.358 |  | p=0.859 |  |  | **p=0.005** |  |
|  | cognitive dysfunction |  | p=0.908 |  | p=0.660 |  |  | p=0.225 |  |
|  |  |  |  |  |  |  |  |  |  |

*Note*: Models adjusted for age, sex, ethnicity, employment status, setting, pre-existing conditions, severity of infection, hospitalisation, intensive care unit admission, COVID-19 variant, vaccination status, duration of Long COVID, and efforts in physical, cognitive, social and self-care activities
